# A perspective in the management of myelomeningocoele in the KwaZulu-Natal Province of South Africa

**DOI:** 10.1007/s00381-020-04506-9

**Published:** 2020-01-23

**Authors:** M. N. Mnguni, B. C. Enicker, T. E. Madiba

**Affiliations:** 1 Department of Neurosurgery, Inkosi Albert Luthuli Central Hospital, University of KwaZulu-Natal, 800 Vusi Mzimela Road, Cato Manor, Durban, 4001 South Africa; 2 Department of Surgery, University of KwaZulu-Natal, Durban, South Africa

**Keywords:** Myelomeningocele, Spina bifida, Hydrocephalus, Management

## Abstract

**Background:**

Myelomeningocoele (MMC) is common in the developing world. The purpose of this study was to investigate the clinical characteristics and management of myelomeningocoele and to identify factors contributing to outcomes.

**Methods:**

This was a retrospective, observational study of consecutive children diagnosed with MMC managed in the Paediatric Neurosurgery Unit at Inkosi Albert Luthuli Central Hospital. Multiple logistic regression analysis identified clinical characteristics, demographics and surgical variables that were associated with outcome.

**Results:**

A total of 309 children were managed during this period (M:F 1.3:1). The most common sites were lumbar, lumbo-sacral and sacral. Mean age at surgical repair was 4.7 ± 15.6 months. Two hundred and eight children had ventriculomegaly, of whom 158 had symptomatic hydrocephalus, requiring CSF diversion. Fifty-eight (21%) patients developed wound sepsis, of whom 13 (22%) developed meningitis (*p* = 0.001). The time to wound sepsis was 9.5 ± 3.6 days. The commonest organism isolated was *Staphylococcus aureus* followed by MRSA. Thirty-two patients (23%) developed shunt malfunction and three (11%) developed ETV malfunction. Twenty children (9%) demised during the admission period. Death was associated with meningitis (*p* < 0.0001), and meningitis itself was associated with wound sepsis (*p* < 0.0001). Hospital stay was 20.4 ± 16 days. Wound sepsis (*p* = 0.002) and meningitis (*p* < 0.0001), respectively, were associated with prolonged hospital stay.

**Conclusion:**

There was a slight male preponderance and hydrocephalus occurred in two thirds of cases. Wound sepsis and meningitis were associated poor outcomes.

## Introduction

Neural tube defects (NTDs) result from failure of neural tube closure that normally occurs at 15–28 days after conception [1]. Myelomeningocele (MMC) is by far the most common and devastating form of NTD, which comprises a sac-like structure that protudes through the vertebral arches and contains meninges, cerebrospinal fluid (CSF) and spinal cord [1, 2].

The incidence of NTD in South Africa (SA) is reported to be 0.77–6.1 per 1000 live births [1, 2], which is slightly higher than the international incidence of 0.17–6.39 per 1000 live births [2–5]. In East Africa, the prevalence of NTDs is estimated to be 1.3 cases per 1000 live births [6]. There are approximately 300,000 new cases of NTDs annually, and 40,000 of these cases were estimated to occur in sub-Saharan Africa [6]. This variation is influenced by racial, geographic and socioeconomic status of the regions surveyed. The incidence of NTDs has previously been under-reported in sub-Saharan Africa due to lack of awareness on the part of parents and limited access to healthcare facilities [6].

The diagnosis of NTD is life-changing for the child, the parents and the community [7]. The condition is associated with CNS abnormalities, resulting in disabilities such as sensory, motor, bladder and bowel dysfunction together with orthopaedic deformities and learning disabilities which can shorten the child’s life-span, and have psychosocial implications for the child including integration into their communities [8]. The optimal management requires a multidisciplinary effort which can be challenging in a resource-limited environment [7].

The literature on MMC is sparse in developing countries like South Africa, which are predisposed to the development of birth defects. The purpose of this paper was to report on clinical characteristics, management and post-surgical outcomes of children diagnosed with MMC in the KwaZulu-Natal (KZN) Province of South Africa. This was achieved by a retrospective observational study of MMC in a tertiary neurosurgical unit based in a developing country.

## Methods and materials

This was a retrospective study of consecutive children with the diagnosis of MMC admitted into the Paediatric Unit in the Department of Neurosurgery at Inkosi Albert Luthuli Central Hospital (IALCH), a tertiary institution located in Durban, the main coastal city of the KZN Province. The Province is located on the east coast of South Africa and has a surface area of 94,361 km^2^ [9].. It has a population of 10 million people of which 3.5 million are under the age of 12 years. Our unit provides the only paediatric neurosurgery service for this Province.

We reviewed the charts of all children with a diagnosis of myelomeningocoele admitted between January 2006 and December 2014. Patient data are kept in a password-protected hospital information management system (Soarian ®, Siemens, USA). The data were analysed for variables such as mode of delivery, demographic profile, clinical presentation, MMC location, presence of CSF leak, age at MMC repair, associated hydrocephalus and management thereof including septic complications associated with MMC repair. The study also assessed the method of CSF diversion and their outcomes as well as length of hospital stay and in-hospital mortality. We excluded patients with incomplete information in their medical files and those who were treated in neurosurgery units outside IALCH. This study was granted approval by the Biomedical Research Ethics Committee (BREC) of the University of KwaZulu-Natal.

The repair of the MMC in our unit involves dissection and reconstruction of the neural placode protecting any functional nerve roots. This is followed by closure of the dura and tension-free closure of myofascial tissue. Complex defects that require flap reconstruction are closed in conjunction with the plastic surgeons. The indication for the insertion of VPS is the presence of active hydrocephalus (HCP). As a policy, we do not insert VP shunts at the same surgical operation as repair of MMC. Patients who developed symptomatic hydrocephalus and were suitable candidates were offered endoscopic third ventriculostomy (ETV).

Statistical analysis was performed using SPSS 21 (Chicago, Illinois, United States of America). Mean and standard deviation was used for categorical variables. Student t test and Mann-Whitney test were used for continuous variables. We assessed the association between time to surgery, presentation with CSF leak and hydrocephalus with wound sepsis and mortality. A *p* value of less than 0.05 was considered statistically significant.

## Results

**Fig. 1:**
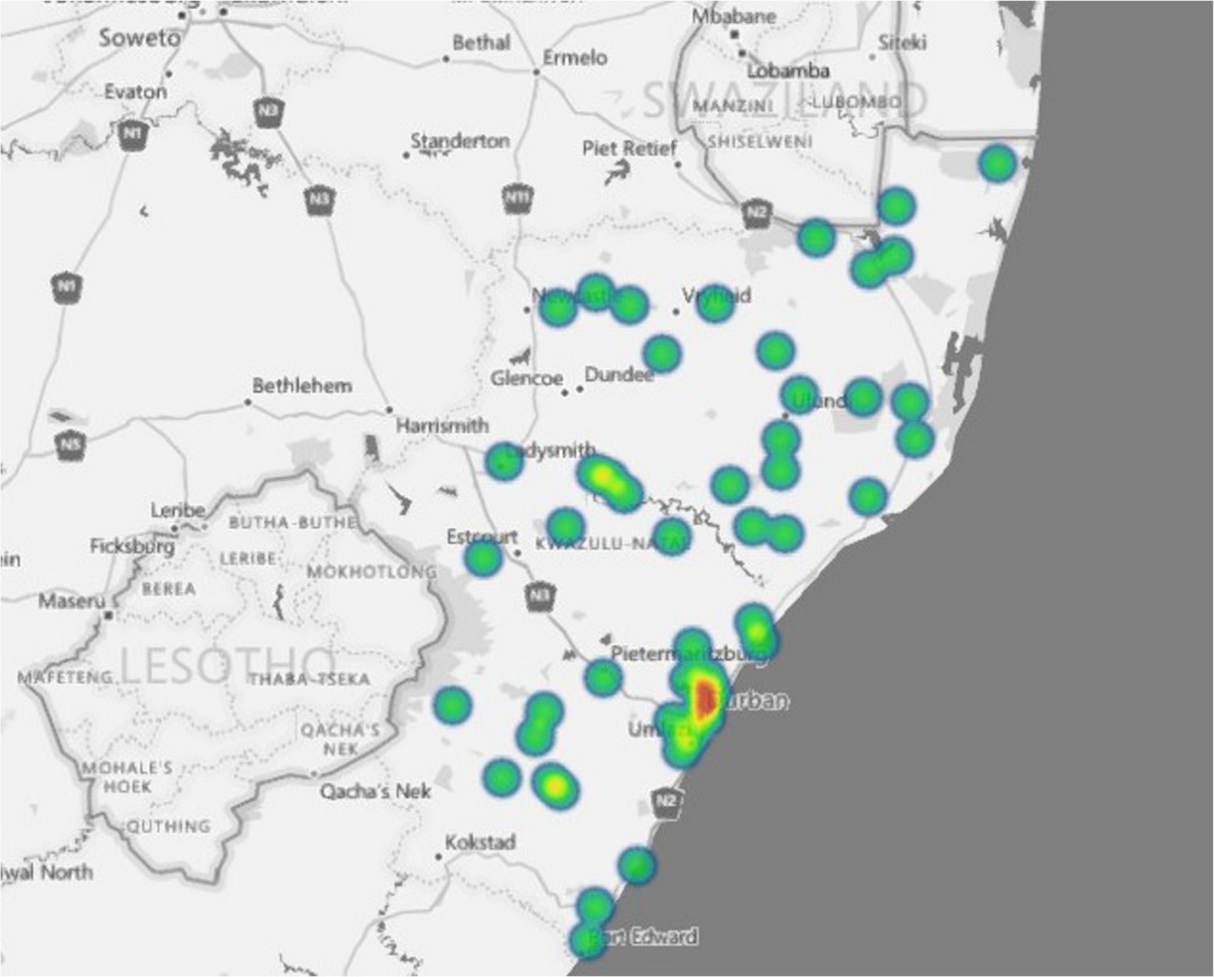
This is a geospatial map showing the regions of KwaZulu-Natal Province from which the children with MMC emanated

A total of 314 children were treated for MMC at our neurosurgical unit during the study period. Five were excluded because of incomplete information in the files, leaving 309 children for analysis. Figure 1 shows geographical distribution of the referral patern of MMC in KZN. There were 173 males (M:F ratio 1.3:1). One hundred and thirty-five children (44%) were delivered via caesarean section and the rest by normal vaginal delivery. Antenatal diagnosis of MMC was possible in only six children (1.9%). Maternal age was documented in only 129 files. Of these,7 mothers (2.3%) were < 18 years of age, 104 were 18–35 years old (33.7%) and 18 (14%) were > 35 years. Only 240 mothers (78%) had attended antenatal clinic; 23 did not attend antenatal clinic (7%) and antenatal clinic attendance was not documented in 46 mothers (15%). There was a family history of MMC in only two mothers; one mother had an older child with MMC and another reported one family member mothering a child with MMC. The majority of MMC were in the lumbar region followed by the lumbo-sacral spine (Table 1). There was CSF leak from the lesion in 156 children (51%) mainly from the lumbar area (Table 1) and 141 children (48%) presented with complete paralysis of the lower limbs.

**Table 1:** Lesion distribution in 309 children with mmc and numbers with csf leak

Site	Total *n* (%)	With CSF leak *n* = 156
Lumbar	175 (56.7%)	83
Lumbo-sacral	72 (23.3%)	43
Sacral	21 (6.8%)	9
Thoracic	17 (5.5%)	7
Thoraco-lumbar	14 (4.5%)	10
Thoraco-lumbo-sacral	5 (1.6%)	3
Cervical	2 (0.6%)	0
Cervico-thoracic	1 (0.3%)	1

Mean age at surgical repair was 4.7 ± 15.6 months. Flap reconstruction was required in 20 children (7.9%). Radiological evidence of ventriculomegaly was seen in 208 children (67%) and symptomatic HCP was observed in only 145 of these (70%). CSF diversion with VPS was necessary in 144 children. ETV was performed as an initial treatment option in one child.

Thirty-two of the 144 patients who underwent VPS (22%) developed shunt malfunction (Table 2). The time to shunt malfunction was 176 ± 83.3 days. Infection was responsible for shunt malfunction in 15 of the 32 children (47%). Nineteen of the 32 children with shunt malfunction underwent revision of the blocked shunt and the other thirteen underwent ETV. In total, therefore, 14 patients underwent ETV. ETV malfunction occurred in three patients after a median 31 days (30–122), all of whom were subsequently managed with VPS insertion; ETV was not reattempted.

**Table 2:** Causes of shunt malfunction in 32 children with myelomeningocoele

Cause	*n*
Infection	15
*Meningitis (12)*	
*Cranial wound dehiscence (leading to shunt exposure) (3)*	
Mechanical failure	14
*Shunt disconnection (10)*	
*Sshunt blockage (reason unknown) (4)*	
Abdominal factors	2
*CSF leakage through abdominal wound (1)*	
*Peritonitis (1)*	
Shunt over-drainage (causing subdural hygroma)	1

Fifty-eight (21%) children developed postoperative wound sepsis, of whom 35 (60%) required surgical debridement, while chemical debridement was sufficient in the rest. The time to wound sepsis was 9.5 ± 3.6 days. The most common organism responsible for sepsis was *Staphylococcus aureus* (Table 3). The presence CSF leak (*p* = 0.20) and/or hydrocephalus (*p* = 0.39) were not independently associated with wound infection. Thirty-four children developed meningitis in the postoperative period (11%).

**Table 3:** Organisms responsible for sepsis in children with myelomeningocoele

Organism	*n*
*Staphylococcus aureus*	11
Methicillin-resistant staphylococcus aureus	8
Acinetobacter baumannii	5
*Klebsiella pneumoniae*	3
Pseudomonas aeroginosa	2
*Escherichia coli*	1
*Enterobacter cloacae*	1
*Enterococcus faecalis*	1
*Candida albicans*	1
*Streptococcus pyogenes*	1

**Fig. 2:**
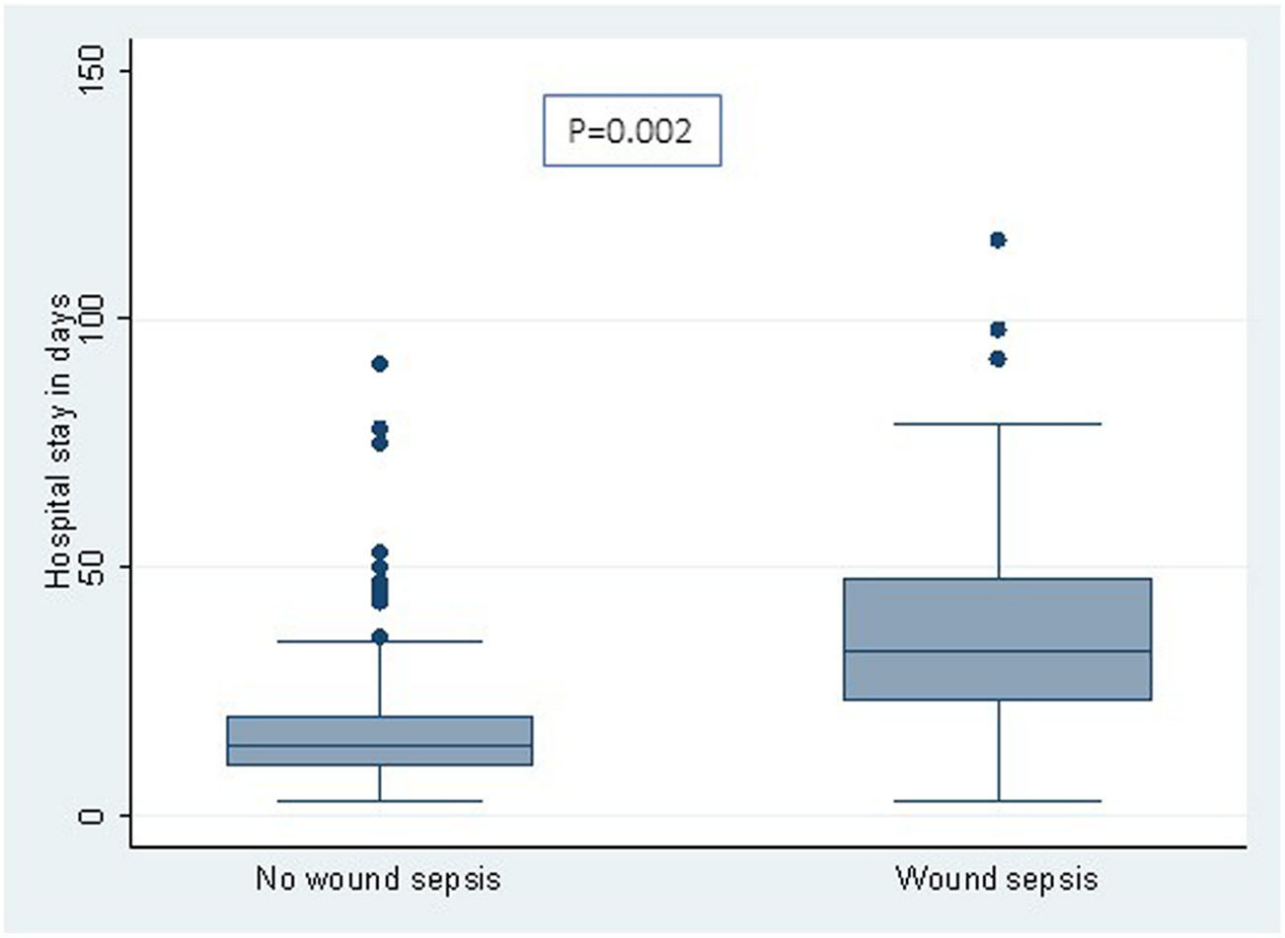
This shows a comparison of the hospital stay in patients with and without wound sepsis. Hospital stay was 38.1 ± 22.3 days for children with wound sepsis and 20.4 ± 16.9 days for those without wound sepsis (*p* = 0.002)

**Fig. 3:**
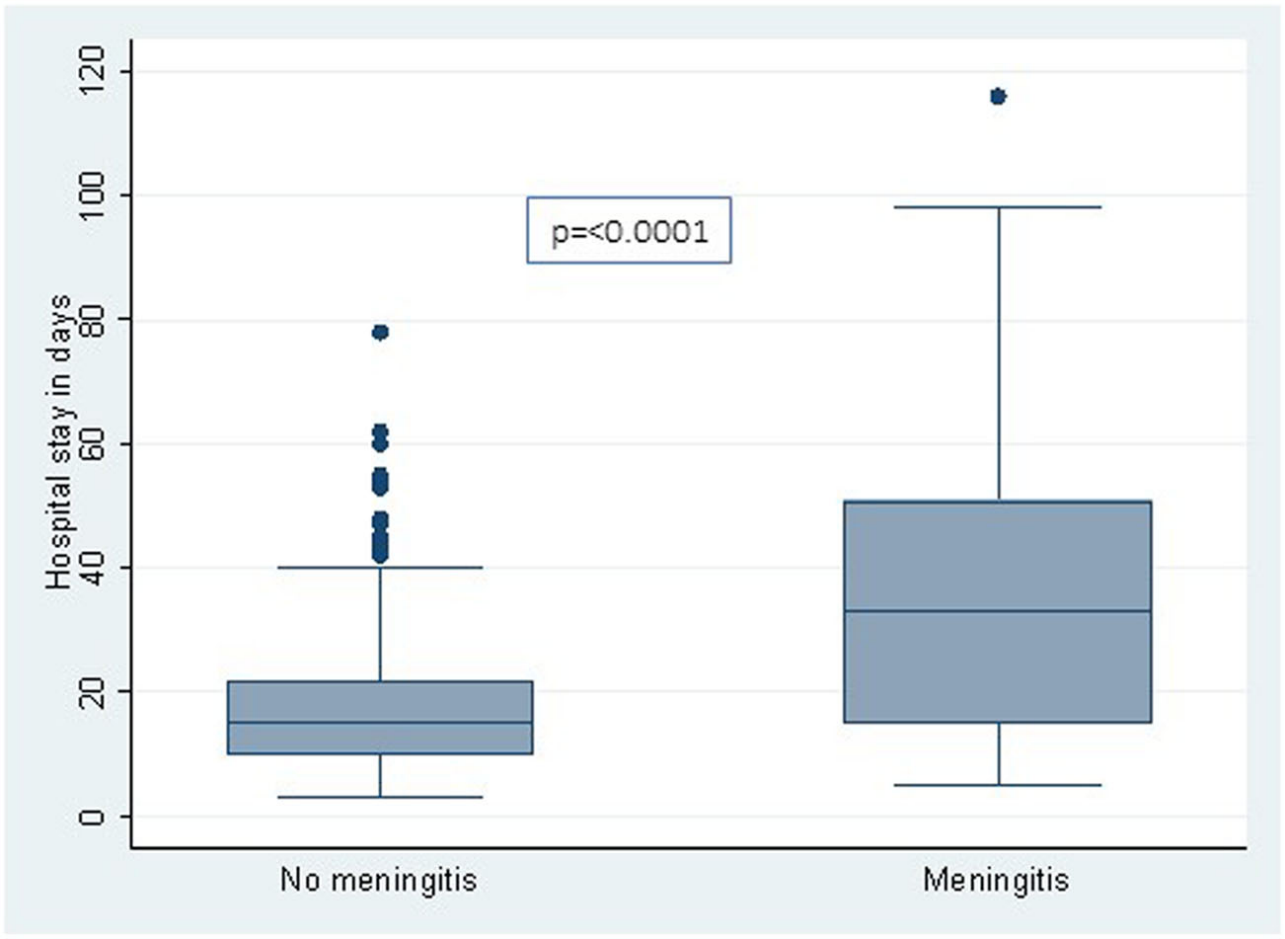
This figure shows a comparison of the hospital stay in patients with and without meningitis. Hospital stay was 40.5 ± 29.7 days for patients with meningitis and 18.4 ± 22.3 days in patients with no meningitis (*p* < 0.0001)

Figure 2 shows the length of hospital stay in children with and without wound sepsis, and Fig. 3 shows comparison of length of hopital stay in children with and without meningitis. Hospital stay was longer for patients with wound sepsis and meningitis. Twenty children (9%) demised during the admission period. Factors influencing mortality, sepsis and meningitis are shown in Tables 4, 5 and 6, respectively. Wound sepsis played a significant role in influencing meningitis (*p* = 0.002), and meningitis was itself a significant factor in influencing mortality (*p* < 0.001). The need for flap reconstruction significantly influenced wound sepsis (*p* < 0.001). Only 54 patients were available for follow-up with the mean follow-up period being 24 + 16.42 months. One patient is known to have died during follow-up.

**Table 4:** Factors influencing mortality

Factor	Present/absent	*n*	Death *n* (%)	*p*
Sepsis	Present	58	7 (12.1%)	0.07
	Absent	218	12 (5.5%)	
	Not stated	31	–	
Hydrocephalus	Present	204	21 (10.3%)	0.26
	Absent	80	6 (7%)	
	Not stated	17	–	
Meningitis	Present	35	12 (34%)	< 0.001
	Absent	251	11 (4.4%)	
	Not stated	21	–	
Gender	Male	173	16(9.2%)	NS
	Female	134	11 (8.2%)	
Plastic reconstruction	Required	20	3 (15%)	0.18
	Not required	250	17 (6.8%)	
	Not stated	37	–	

*Not all had the factors recorded in the records

**Table 5:** Factors influencing wound sepsis

Factor	Present/Absent	n	Wound sepsis n (%)	p
Hydrocephalus	Present	188	37 (19.7%)	0.39
	Absent	82	18 (22%)	
	Not stated	37	–	
CSF Leak	Present	151	35 (23.2%)	0.20
	Absent	125	23 (18.4%)	
	Not stated	31	–	
Plastic reconstruction	Required	20	12 (60%)	< 0.001
	Not required	247	43 (17.4%)	
	Not stated	40	–

**Table 6:** Factors influencing meningitis

Factor	Present/absent	*n*	Meningitis *n* (%)	*p*
Wound sepsis	Present	58	13 (22.4%)	0.002
	Absent	219	16 (7.3%)	
	Not stated	30	–	
CSF leak	Present	153	17 (11.1%)	0.39
	Absent	132	17 (12.9%)	
	Not stated	40	–	

## Discussion

Observations made from this study are similar to those in the international literature with a few exceptions. There was a slight but not clinically significant male preponderance. The occurrence of the MMC sac in the lumbar, lumbo-sacral and sacral areas is in keeping with the international literature where the common sites of occurrence are the lumbar and lumbosacral areas [10–12]. HCP was noted in 67% in this series which falls within the 51–80% reported in the literature [4, 13–16].

Surgical repair of the MMC is generally performed within 24 h after birth to minimize the risk of infection and to preserve existing function in the spinal cord [7]. Most authors [11, 17] concur that a surgical closure longer than 48 h after delivery is an important risk factor for wound sepsis and other complications, while others contend that the timing of MMC repair has no influence on complications [15–17]. In our setting, patients are referred for repair long after birth, which explains the late age of repair of 4.7 months in this study.

The delay in surgical repair of these lesions is not ideal or standard of care. The constraints of healthcare resources are the key factors resulting in these undesirable delays in our setting. In children with delayed presentation, the neural placode is destroyed and overgrown by granulation tissue. At surgical repair, the granulation tissue is resected, the dura is closed in watertight fashion and the myofascial tissue is mobilized to achieve a tension-free closure [18]. One cannot determine with certainty whether any neural tissue is functional at this stage, but the principle is to preserve identifiable neural tissue [18].

VPS insertion remains the gold standard for the treatment of HCP in children with MMC. However, it is not without complications. It is known to have a significant failure rate, particularly related to infective complications [10, 19]. Furthermore, these children already have vulnerable CNS tissue and the development of additional infection further worsens cognitive function and other outcomes. This has made the timing of VPS insertion an area of debate [19]. Whereas some authors suggest that VPS insertion at the same time or within a week of myelomeningocele closure increases the risk of shunt infection [10, 15, 20], others report no significant difference in the rate of shunt infection and dysfunction [21]. Yet other studies report that a delay in MMC repair surgery significantly increases the risk for ventriculitis and meningitis [22]. Our Unit has adopted a policy of employing delayed insertion of VP shunt, especially in the presence of a leaking MMC, the rationale being to limit the rate of infection.

Surgical site infection is a frequent complication in myelomeningocele repair [12]. The prevalence of wound sepsis in this series was 21% which falls within the 12.8–34% reported in the literature [11, 12, 16, 23]. Risk factors for surgical site infection are delayed presentation, size of defect, length of operative procedure and the use of flap reconstruction [11, 12]. Similarly in this study delayed presentation and use of surgical flap reconstruction contributed towards a high rate of surgical site infection. Although the source of sepsis following MMC repair varies in the literature [12], the profile of microorganisms isolated in our patients are similar to other reported series [11, 12, 24]. Demir et al. noted that cultured microorganisms in children with MMC were of nosocomial origin and resistant to antibiotics [11].

Shunt malfunction developed in 22% in this series which falls within the 15–45.9% reported in the literature [25–27]. The common causes of shunt malfunction are shunt blockade (11–20%) [11, 16, 26, 27], shunt infection (3.5%) and shunt migration (0.9%) [26, 27]. Risk factors for the development of meningitis include CSF leakage from the MMC, and complex defects requiring flap transposition [11, 12, 16]. The 11% meningitis rate in this series was lower than the reported 19–21% [11].

Our in-hospital mortality rate was 9%. This is higher than the 5–7.5% mortality rate reported by in the international literature [10, 28]. This study also showed meningitis and wound sepsis to be significant factors influencing mortality and length of hospital stay. The need for flap reconstruction also indirectly contributed to increased hospital stay. This is supported by Demir et al., who have shown that meningitis, VP shunt infection and surgical wound-site infection prolong the duration of hospitalization and increase hospital costs [11].

The advent of in-utero repair of MMC [29] is a welcome development. Where fetal surgery is considered, prenatal evaluation should include high resolution ultrasound, magnetic resonance imaging and amniocentesis [30]. However, this form of treatment is not routinely performed in developing counties [3]. Only 2% of MMC were diagnosed in the prenatal stage; this is despite 78% of the mothers having attended antenatal care clinics. This is in contrast to the findings in the world literature where antenatal diagnosis can be as high as 60% [31]. This low rate of prenatal diagnosis may be explained by the lack of screening tools and expertise in most antenatal care centres in our setting. This leaves many of these congenital defects undetected until delivery. Furthermore, performing an anatomical scan at 18–23 weeks requires appropriate training in the use of a 3D scanner [2]. Such resource is only available in IALCH,which is the only referral centre for high-risk maternal cases. We are in agreement with other authors that the late or no presentation for antenatal care and the low antenatal diagnosis rate in developing countries may be related to the lack of awareness of the population regarding this condition [32, 33].

The South African Government has put emphasis on primary healthcare, which include antenatal care clinics [29]. This will allow in utero diagnosis of NTDs which should be accompanied by counselling with regard to fetal surgery or termination of pregnancy as provided for by the Choice on Termination of Pregnancy Act No. 92 of 1996 [34]. Despite advances in the treatment of MMC, no treatment exists that will completely eliminate the serious disability or premature mortality associated with it. Thus managing children with MMC in sub-Saharan Africa can be potentially devastating due to prevailing low socioeconomic status, inadequate health facilities and other factors [35].

This study does have some limitations. These include the fact that the design was retrospective, and not all data were available for analysis. However, it reflects a large number of the burden of MMC in a South African institution serving a large population.

In conclusion, myelomeningocele is one of the main contributors of burden of disease affecting children managed in a Neurosurgical unit in KZN. There was a slight male preponderance and hydrocephalus was present in two thirds of cares. Myelomeningocoele was associated with significant morbidity. Wound sepsis and meningitis were associated with prolongation of hospital stay. The need for flap reconstruction together with resultant wound sepsis as well as meningitis were associated poor outcomes.
